# Care-Seeking for Diarrhoea in Southern Malawi: Attitudes, Practices and Implications for Diarrhoea Control

**DOI:** 10.3390/ijerph13111140

**Published:** 2016-11-15

**Authors:** Salule Masangwi, Neil Ferguson, Anthony Grimason, Tracy Morse, Lawrence Kazembe

**Affiliations:** 1 Centre for Water, Sanitation, Health and Appropriate Technology Development, University of Malawi, The Polytechnic, P/B 303, Chichiri, Blantyre 3, Malawi; tonygrimason@yahoo.com (A.G.); tracythomson@africa-online.net (T.M.); 2 Department of Mathematics and Statistics, University of Malawi, The Polytechnic, P/B 303, Chichiri, Blantyre 3, Malawi; 3 Environmental Health, Department of Civil Engineering, University of Strathclyde, Glasgow G4 0NG, UK; n.s.ferguson@strath.ac.uk; 4 Scotland Chikwawa Health Initiative (SCHI), P.O. Box 30376, Blantyre 3, Malawi; 5 Department of Statistics and Population Studies, Faculty of Science, University of Namibia, P/B 13301, Windhoek, Namibia; lkazembe@yahoo.com

**Keywords:** care-seeking behaviour, diarrhoea, multilevel modelling, Southern Malawi

## Abstract

This paper examined care-seeking behaviour and its associated risk factors when a family member had diarrhoea. Data was obtained from a survey conducted in Chikwawa, a district in Southern Malawi. Chikwawa is faced with a number of environmental and socioeconomic problems and currently diarrhoea morbidity in the district is estimated at 24.4%, statistically higher than the national average of 17%. Using hierarchically built data from a survey of 1403 households nested within 33 communities, a series of two level binary logistic regression models with Bayesian estimation were used to determine predictors of care-seeking behaviour. The results show that 68% of mothers used oral rehydration solutions (ORS) the last time a child in their family had diarrhoea. However, when asked on the action they take when a member of their household has diarrhoea two thirds of the mothers said they visit a health facility. Most respondents (73%) mentioned distance and transport costs as the main obstacles to accessing their nearest health facility and the same proportion of respondents mentioned prolonged waiting time and absence of health workers as the main obstacles encountered at the health facilities. The main predictor variables when a member of the family had diarrhoea were maternal age, distance to the nearest health facility, school level, and relative wealth, household diarrhoea endemicity, and household size while the main predictor variables when a child had diarrhoea were existence of a village health committee (VHC), distance to the nearest health facility, and maternal age. Most households use ORS for the treatment of diarrhoea and village health committees and health surveillance assistants (HSAs) are important factors in this choice of treatment. Health education messages on the use and efficacy of ORS to ensure proper and prescribed handling are important. There is need for a comprehensive concept addressing several dimensions of management and proper coordination of delivery of resources and services; availability of adequate healthcare workers at all levels; affordability to accessibility of healthcare resources and services to all communities; acceptability and quality of care; intensification of health education messages on the use and management of ORS, and prompt and timely treatment of diarrhoeal illness.

## 1. Introduction

Diarrhoea is a significant cause of morbidity and mortality in Malawi. In 2013, diarrhoeal disease was reported to have been one of the highest causes of premature deaths in Malawi [1]. This concurs with an earlier survey undertaken in 2005 which reported that the national prevalence of diarrhoea was 22% [2] and that diarrhoeal disease was the cause of approximately 1 in 5 (19%) deaths in children under the age of five [3].

Prevention and reduction of morbidity and mortality are among the priority goals outlined in the Millennium Development Goals (MDGs) 4, 5 and 6 and in the UN Millennium Declaration—adopted in September 2000 by the United Nations and endorsed by 189 countries with the main objective of improving people’s lives [4].

In Malawi, the control of communicable diseases, through treatment and reduction of morbidity and mortality, is delivered primarily through the Primary Health Care (PHC) system, which is the foundation of Malawi’s Health Policy [5]. The PHC is delivered through the Essential Health Package (EHP) [5]—the primary goal of which is to ensure that health services are accessible to all Malawians. The rationale behind the EHP as a pro-poor strategy is to focus on the major causes of morbidity and mortality, and address medical conditions and service gaps that disproportionately affect the rural poor [6]. Emphasis in PHC system is on prevention and effective treatment of cases through health surveillance assistants (HSAs) and village health committees (VHCs) who are mostly responsible for awareness, monitoring, and supervision of preventive measures in rural communities and development partners such as non-governmental organisations (NGOs) who are involved in health education messages, distribution of healthcare resources and monitoring [5].

Information on the utilisation of health care at PHC and health seeking behaviour of health beneficiaries has important policy implications. Previous studies have observed that factors that influence the type and quality of treatment when diarrhoea symptoms occur include socioeconomic, community social and infrastructure networks, beliefs and household decision making processes [7,8]. The cost of health services has also been observed to be a determinant of health seeking behaviour [7,8]. Other factors that have affected care-seeking behaviour include knowledge and duration of sickness, judgement of the seriousness of sickness [7], accessibility to health facilities, and demographic characteristics [9]. The most common and effective response to dehydration caused by diarrhoea in Malawi is the intake of oral rehydration therapy (ORT) which is promoted through commercially prepared oral rehydration solution (ORS), facility-based provision of premixed ORS and various home-made grain-based rehydration fluids [1].

This paper examined the pattern of health seeking behaviour and associated risk factors among households in Southern Malawi when symptoms of diarrhoea were identified in a child or a family member. In particular, the paper: (i) investigates actions taken by households when a child or a family member was perceived to be suffering from diarrhoea; (ii) determines the socioeconomic differentials of households in patterns of utilization of diarrhoea treatment services; (iii) examines the impact of PHC system on households’ health seeking behaviour; and (iv) proposes how the findings of this study can be utilised to improve case management of diarrhoea.

## 2. Background

Chikwawa is a district in Southern Malawi. It has a surface area of 4755 km^2^ and an elevation of only 100 m above sea level. Out of a population of about 470,000, 22% are women of childbearing age. 

The climate is subtropical, with a rainy season that runs from November through April. There is little to no rainfall throughout most of the district from May to October. Chikwawa has an average monthly temperature of 28.4 °C, with a minimum of 15.2 °C and a maximum of 45.6 °C [2]. It is normally hot and humid in the months of November to April and hot, dusty and very dry in the months of July to November. Average rainfall in Chikwawa is around 915 mm/year, mostly falling in November–March [2]. Malawi’s biggest river, which drains Lake Malawi and is characterised by big marshes, passes through this district. Chikwawa is also home to Malawi’s biggest sugar plantation and two national game reserves.

Because of these climatic and geographical features, Chikwawa is faced with a number of environmental and socioeconomic problems. Almost every year, Chikwawa is faced with floods from the Shire River and others that feeds into it. The flooding sweeps away crops and livestock thereby creating food insecurity responsible for malnutrition to many children. They also sweep away human dwellings, displacing hundreds of people. Floods leave behind marshes and other pools of water. These marshes and pools of water can be unsafe water sources (to poor families without access to improved water sources) that are subject to contamination from both point sources e.g., indiscriminate faecal littering of the environment by humans who either do not have access to a latrine or would prefer not to use a latrine and domestic animals which roamed freely and diffuse pollution e.g., surface run-off from land into rivers, streams etc. during the rainy season and flooding events [10]. Currently diarrhoeal morbidity (percentage of people who had diarrhoea in the two weeks preceding the 2004 Malawi Demographic and Health Survey) in the district is estimated at 24.4% [11]. This is statistically higher than the national average of 22% for diarrhoea morbidity [2].

## 3. Methods

### 3.1. Sample

The survey was conducted in Chikwawa in 2007, which has a total of more than 400 villages (communities). The study used a survey methodology similar to that used in national surveys [12] to produce a district representative sample of communities, households and individuals. Enumeration maps from the Malawi National Statistical office were used as a guide in sampling the villages. These enumeration maps are used for professional national surveys such as the Demographic Health Surveys (DHS) [2]. The survey was carried out under the umbrella of the Scotland-Chikwawa Health Initiative (SCHI), a Scottish Government funded non-profit organisation.

Ten enumerators were recruited amongst those that had already been involved in national surveys at the National Statistical Office in Malawi. The SCHI provided a supervisor who is an experienced health surveillance assistant in Chikwawa district and was previously involved in a number of surveys and data collection.

The enumerators were given one week intensive training on the questionnaire and had two days of a pilot study in order to: (a) acquaint themselves with the questions; (b) afford them an opportunity to ask questions, seek clarifications, and make general questions where necessary; (c) accustom them with survey, interviewing, and house selection techniques. The exercise also sought to familiarise them with the problems they were going to encounter in the field and at the same time share their previous experiences in such exercises. Since the questionnaire was translated in a local language, the pilot study was also meant to clear any ambiguities and problems in its interpretation and administration.

A two-stage survey methodology was adopted to produce a district representative sample of households. The first-stage involved sampling of villages that were strategically selected with a probability proportional to the number of enumeration areas in each Traditional Authority. Chikwawa has eleven traditional authorities and each traditional authority has several villages under its jurisdiction. The second sampling stage took place on the day of interviews. Households were systematically chosen with equal probability sampling. Only the most senior woman from each household was eligible for interviews and all other members of the households were asked to leave the interviewing premises to avoid interference.

Sample sizes in the selected households ranged from 1 to 13. After discarding missing and uncompleted information for 7 households, data for a total number of 1403 households nested within 33 villages was obtained for this study.

Permission to conduct the survey was received from the Malawi National Health Sciences Research Committee (MNHSRC), the District Commissioner for Chikwawa District and traditional leaders.

### 3.2. Variables

#### 3.2.1. Outcome Variables

Distribution of outcome variables is listed in Table 1. The most senior woman in each household was first asked to explain the actions they most likely take when a member of their household (excluding children under the age of five) has diarrhoea. There were four responses: (a) visiting a health facility; (b) home treatment using oral rehydration solution (ORS) previously obtained from health facility; (c) home treatment using ORS bought from a shop, vendor or open market; and (d) home use of other solutions fluids or doing nothing. A series of dichotomous variables were formed for each response by coding 1 for a given response and zero otherwise.

A mother (the most senior mother) was then asked to explain exactly what they did last time when a child (five years or less) in the family had diarrhoea. Answers were put into four categories: (a) visited health facility; (b) administered ORS; (c) administered homemade solutions or other fluids and (d) did nothing. Notice that the response categories for “a member of the household with diarrhoea” and for “a child in the family with diarrhoea” are different because no single respondent said they do nothing or that they use traditional methods when a member of the family had diarrhoea. Similarly the responses are different on use of ORS because respondents specifically mentioned either ORS bought from shops/vendors or ORS obtained from a health facility when asked about what they do when a family member has diarrhoea. On the other hand, most respondents only remembered using ORS to treat a child last time the child had diarrhoea but could not specifically remember if the ORS was either bought from shops/vendors or was obtained from health facility.

In this study a child was defined as a person with the age of 5 years or below. Considering that diarrhoea morbidity is very high in Chikwawa and that adults are also highly vulnerable this study also asked about care practices for all family members rather than restricting the analysis to children. In addition the study wanted to compare possible differences in the types of actions taken when a child had diarrhoea to a general case of a family member who had diarrhoea.

The respondents were also asked to explain the time it takes for them to treat any member of their household who has diarrhoea. There were three responses: (a) same day; (b) one day; and (c) two or more days. The questions also sought to identify the problems householders normally encountered: (i) to reach the nearest health facility and (ii) at the health facility.

#### 3.2.2. Explanatory Variables Included in the Models

Highest maternal school was categorized into three groups: no formal school, primary school, and secondary and/or tertiary school.

Relative wealth was derived by analysing household possessions, quantity of animals and birds and type and quality of house. The method of “variations” [13] that assigns weights to indicator variables and uses the inverse of the proportion of number of households with an asset or service as the weight for the indicator was used. The principle behind this procedure is that the costlier an item, the wealthier a household needs to be to possess one, giving the highest weights to the least possessed assets. Caution was taken to ensure that problems arising with this weighting scheme in certain assets, such as motorcycles, that are rare, but are not as costly as a car, were either excluded or were included amongst items closer in function and quality. A categorical variable was derived by cutting the wealth index distribution into three distinct segments based on observed clusters such that the first segment is from households with indices 0 to less than 0.003; the second segment is from 0.003 to less than 0.01, the third segment is from 0.01 and above.

Distance to the nearest health facility was categorized in three groups: less than one kilometre, one to two kilometres, and more than two kilometres.

HSAs (health surveillance assistants) were coded 1 if available and 2 if not available.

Maternal age (the age of the most senior woman in the household) was a continuous variable.

Household diarrhoea endemicity (HH DRR) was obtained by dividing the total number of reported diarrhoea episodes over a period of eight months by the total number of members in a household. This is a continuous variable.

Household size is the total number of members in a given household and this was taken as a continuous variable.

## 4. Analysis and Estimation

The data being analysed in this study has two levels, i.e., household and community levels. In order to quantify variation in care seeking behaviour between communities multilevel modelling was utilised to analyse the data with households as level 1 and communities as level 2. A series of two-level binary logistic regression models with any action taken when a member of household is perceived to have diarrhoea as response variables were constructed to test their pattern of variation and corresponding predictors.

The binary regression model [14,15,16] was used to explain the probability of outcomes for households. If the *i*_th_ household from the *j*_th_ village had the required attribute then a response would be written as:

$$y_{ij}=\{\begin{array}{l} 1\;\mathrm{if}\;\mathrm{the}\;i_{\mathrm{th}}\;\mathrm{household}\;\mathrm{from}\;\mathrm{the}\;j_{\mathrm{th}}\;\mathrm{community}\;\mathrm{had}\;\mathrm{the}\;\mathrm{required}\;\mathrm{attribute}\\ 0\;\mathrm{otherwise} \end{array}$$

such that: $y_{ij}|\pi_{ij}=Ber\left(\pi_{ij}\right)$ and $\log it\left(\pi_{ij}\right)=x_{ij}\beta +u_{0j}$ is a random components model. *i* = 1, …, *I_j_* households and *j* = 1, …, *J* communities, with $\pi_{ij}$ as the probability that the *i*_th_ household in the *j*_th_ community had the attribute. The vector β is a regression coefficient corresponding to a covariate *x_ij_*.

Variation at the community level is modelled through *u*_0*j*_ such that $u_{0j}–N\left(0,\sigma_{u}^{2}\right)$.

Considering the complexity and high-dimensional nature of the models being used which can make it difficult to find solutions analytically, estimation was performed using Bayesian procedures available in the MLwiN 2.10 software (University of Bristol, Bristol, UK). Initial estimates to obtain prior samples were derived using second-order penalised quasi-likelihood (PQL) procedures with restricted iterative generalised least-squares (RIGLS; [17]). Stability of all model parameters was monitored by observing the Raftery-Lewis and the Brooks-Draper mean diagnostics in MLwiN 2.10 [18]. The maximum number of iterations performed to achieve stability was 80,000.

## 5. Results

Distribution of response variables is summarised in Table 1. When women were asked what action was taken when a family member suffered from diarrhoea the majority of women (67%) reported that they visited a health facility. Approximately 1 in 4 women stated that they prepared and administered ORS which was provided either free-of-charge by a health facility (16%) or purchased from nearby shops, market or vendors (11%). A small percentage of respondents (7%) stated that they administered “other fluids” such as salt and sugar solutions at home. When asked what action was taken the last time that a child within the family suffered from diarrhoea 13% stated that they took the child to a health facility, while the majority (68%) stated that they administered either health facility acquired or homemade ORS and 14% mentioned that other fluids were administered. One in twenty women (5%) stated that they did nothing other than keep a watchful eye over the child until the illness subsided.

When questioned on the time taken to treat a family member of the household after the onset of symptoms most women (85%) stated that they administered treatment the same day, while one in every ten women stated that they waited 24 h before doing so. Approximately one in twenty (6%) stated that they would wait two or more days before administering treatment.

Three out of four women stated that (73%) long travelling distances and/or transport costs were the most significant problems they encountered to reach their nearest health facility, 7% highlighted the demands of work (domestic and agricultural) and 8% mentioned other problems. When questioned about problems encountered after reaching the health facility 73% mentioned prolonged waiting times and non availability of health workers, 35% mentioned lack of drugs, 12% mentioned cost of medical services, and 8% mentioned other reasons.

Table 2 shows descriptive statistics for predictor variables on diarrhoea knowledge. One in three women (35%) live within 1 km reach of a health facility, 42% between 1 to 2 km and 23% reside more than 2 km away from nearest health facility. The youngest respondent was 15 years old, the oldest was 89 years and on average the respondents were 35 years old with a median of 31 years.

Multilevel binary logistic regression results of care-seeking behaviour when a member of the household suffered from diarrhoea are given in Table 3. Highest school level for matriarchal figures, maternal age, household diarrhoea endemicity, and household size were the main predictor variables for those that visit a health facility when a member of their family suffered from diarrhoea. Mothers that had attended primary or secondary school were less likely to have taken those who were suffering from diarrhoea to a health facility when compared to those without any formal education (OR = 0.698; 95% CI: 0.538, 0.914 and OR = 0.387; 95% CI: 0.230, 0.650; respectively). Older mothers, large households, or families with high diarrhoea endemicity were also less likely to have taken members of their households to a health facility when they had diarrhoea illness.

Distance and household diarrhoea endemicity were the only two significant predictors for those that administered ORS obtained from a health facility when a member of their household was suffering from diarrhoea. Those that live more than 2 km away from the nearest health facility were less likely to have used ORS than those that lived within a kilometre of a health facility (OR = 0.477; 95% CI: 0.225, 1.010; *p* = 0.053). High diarrhoea endemicity was positively related to the administration of ORS previously obtained from a health facility.

Use of ORS bought from shops or market had two significant predictors: school level and relative wealth. Mothers who attended primary or secondary school were more likely to use ORS bought from an open market to treat a member of the household when compared to mothers who had not attended any formal school. Households with average wealth and the wealthiest were more likely to have used bought ORS than the poorest families in the communities.

Relative wealth, distance to the nearest health facility, maternal age, and household size were significant predictor variables for the administration of “other fluids” or “solutions” at home. Households with relatively average wealth and the wealthiest were less likely to have used other fluids or solutions when compared to very poor families. Families living 1 to 2 km away from a health facility were more likely to have used other fluids or solutions than those that lived within a kilometre from a health facility (OR = 1.840; 95% CI: 1.010, 3.320). Similarly households that were more than 2 km away from a health facility were marginally more likely to have used other fluids or solutions than those within a kilometre from a health facility (OR = 1.733; 95% CI: 0.878, 3.387; *p* = 0.11). Older mothers and large households were also more likely to have used other fluids or solutions.

There was variation between communities for those that had visited the health facility ($\sigma_{u}^{2}$ = 0.28; 95% CI: 0.025, 0.54) and those that had used ORS previously obtained from health facility ($\sigma_{u}^{2}$ = 0.38; 95% CI: −0.01, 0.75; *p* = 0.053). There was no statistical evidence at either 5% or 10% significance level of any variation between communities for those that administered bought ORS and those that administered other fluids or solutions.

Table 4 show multilevel binary logistic regression results for care-seeking behaviour when a child had suffered from diarrhoea disease. Maternal age, distance to a health facility, and existence of a VHC were significant predictor variables for mothers who visited a health facility when a child had diarrhoea. Older mothers were unlikely to have visited a health facility (OR = 0.980; 95% CI: 0.970, 1.000; *p* = 0.018). Mothers from communities that are more than 2 km away from the nearest health facility were marginally more likely to have visited a health facility (OR = 2.054; 95% CI: 0.942, 4.482; *p* = 0.07). Similarly children from communities without a VHC were more likely to have been taken to a health facility.

Existence of a VHC, distance to health facility and household size were the predictor variables for those that administered ORS when a child had diarrhoea. Mothers in communities without a VHC were more unlikely to have administered ORS. Similarly families living more than 2 km away from the nearest health facility were unlikely to have administered ORS at home. However, children from large households were marginally more likely to have been given ORS at home when they had diarrhoea (OR = 1.072; 95% CI: 0.990, 1.150; *p* = 0.08).

Mothers who resided in communities located 1 to 2 km and more than 2 km away from a health facility were more likely to have given their children other fluids or solutions when their children had diarrhoea. Older mothers were also more likely to have used other fluids or solutions to treat their diarrhoea children (OR = 1.020; 95% CI: 1.000, 1.030; *p* = 0.015).

Formal education and maternal age were the only significant factors that influenced mothers’ lack of action against diarrhoea when a child had diarrhoea. Mothers who attended at least a secondary school were marginally less likely to have done nothing while older mothers were more likely to have done nothing when children in their households suffered from diarrhoea. There was no evidence of any community differences on visiting a health facility, using ORS, using other fluids or solutions and doing nothing when a child had diarrhoea.

## 6. Discussion

This study was developed to obtain the pattern of variation and factors associated with care-seeking behaviour when a member of a household suffered from diarrhoea. Responses for a total of 1403 women were analysed and whose summary measures are included in Table 1. It was evident that there are clear discrepancies between reported action normally taken when a member of the family had diarrhoea illness and the last action that was actually taken when a child suffered from diarrhoea disease. The majority of women (67%) stated that they normally visited a health facility when a member of the family suffered from diarrhoea illness. Very few reported that they administered ORS or other fluids at home. However, the response changed when asked to explain exactly what they did last time a child had suffered from diarrhoea disease in the family. The majority (68%) administered ORS to the children while only13% actually visited the health facility and 14% administered other fluids or solutions at home.

ORS is normally administered at home by families when a child or a member has diarrhoea and is distributed free of charge through health facilities, Village Health Committees (VHCs) and Health Surveillance Assistants (HSAs). When someone is perceived to have diarrhoea, families often utilise existing stocks of ORS within the household before seeking fresh supplies—some of which may be time expired. Overall, the statistics are not significantly different from national results of 2004 where 61% of the children with diarrhoea received ORS [2].

In this study a large number of women highlighted the long distance they had to travel and transport costs involved (73%) as the major problems they encountered to access their nearest health facility. This is slightly greater than the national average (63%) of 2004 [2]. Chikwawa is a low altitude area and pathways within and between villages are usually comprised of mud, rocky or swampy paths which become impassable during the rainy season. Some villages are located up to 5 km from the nearest motorised road. Having covered this distance with a child that is ill there is still no guarantee of a means of transportation to transport them to the nearest health facility. In this study one in three women (35%) lived within 1 km reach of a health facility, 42% between 1 to 2 km and 23% resided more than 2 km away from nearest health facility. The study also showed that those that lived longer distances from health facilities were less likely to use a health facility ORS and instead were more likely to have used other fluids or solutions to treat diarrhoeal illness amongst household members. The transportation issues encountered by the rural communities in this study resulted in less health facility visitations—hence less opportunities in accessing healthcare resources such as ORS. These problems are accentuated for the elderly, infirm and disabled people who reside within such communities.

Complaints of prolonged waiting times at health facilities before being seen by a health worker, health workers failing to show up for work and shortage of drugs are common in Malawi, especially in rural areas such as Chikwawa, which like other Districts in Malawi, suffers from a severe shortage of health workers at all levels, making access to knowledgeable health professionals and appropriate treatment unreliable. In addition, the use of health facility staff for specific campaigns (e.g., measles vaccinations, cholera prevention etc.) often takes staff away from their general duties and can reduce their availability even further [19]. With the exception of district hospitals, other health facilities in Chikwawa (as in other rural areas of the country) struggle to attract qualified medical and other allied health professional staff because of the remoteness of the area and lack of social and domestic facilities.

The regression results on actions normally taken when a family member has diarrhoea reveal interesting results. Mothers who attended at least primary school education were unlikely to have taken a member of their family to a health facility to be treated for diarrhoea and were more likely to have purchased ORS from a nearby market. This finding is similar to those of other investigators [11,20,21,22] who observed that “educated” people appear to have a better understanding of the cause and treatment of diarrhoea i.e., recognising the need to administer ORS treatment immediately and not necessarily delay seeking treatment from a healthcare facility. Wealthier households in the communities were more likely to have purchased ORS and were less likely to have used other fluids or solutions for the treatment of diarrhoea. Such households probably have a greater a source of disposable income to purchase ORS supplies and begin treatment immediately to avoid any unnecessary delay. Less wealthier households may delay ORS administration as a result of the travelling time required to access a health facility which provides ORS free of charge on prescription.

Although instructions for preparation of ORS solutions are provided on packets of ORS this may not be helpful in Chikwawa where almost half of the women interviewed had not attended any formal school and many are illiterate. This situation is exacerbated when the written instruction of the packet are in English and not Chichewa, the vernacular language of the country. Previous studies [23] have shown that drinking water stored in rural households is often contaminated with bacterial faecal indicators of pollution. Therefore if the water is not boiled prior to the preparation of the ORS solution the use may be counterproductive. In rural areas it is common for women to prepare ORS solutions using either partially heated water or untreated water. More research is needed in Chikwawa to assess handling and preparation of ORS and to observe behaviour when there is diarrhoeal illness.

Older mothers were less likely to recommend or visit the nearest health facility and were more likely to use other fluids or solutions. They were also more inclined to use traditional methods of treatment when a family member has diarrhoea, essentially calling upon their years of experience in childcare and diarrhoea management. Similarly members from large households were less likely to visit health facilities but were more likely to be treated with home-made fluids or solutions. Large households may also have experience in the management of diarrhoea and may find it easier treating the disease using home-made remedies. Household diarrhoea endemicity variable was included to examine if the magnitude of diarrhoea in the households has any bearing on care-seeking behaviour. Residents from high endemic households were less likely to visit health facilities but were more likely to use ORS previously obtained from HSAs or a health facility [24]. Frequent diarrhoea occurrences may compel households to stock ORS from healthy facilities, which may later be used to treat diarrhoea cases at home.

Variation between communities for those that visited health facilities and those that used ORS previously obtained from a health facility may explain differences in health service delivery in the communities. Health service delivery system in Malawi is in four categories that include community, primary, secondary and tertiary care levels [25,26]. At the community level, service is provided through HSAs and VHCs. The focus is on preventative interventions. Primary care is delivered through clinics and health centres. District and central hospitals provide secondary and tertiary care services, respectively. Communities that were included in this study were drawn from different areas that have access to different categories of health service delivery systems. The quality of health delivery depends on the quality of human and material resources at these different levels of health care delivery. In most cases communities near district and rural hospitals are more likely to have better services than communities near health centres. Medical supplies and equipment are normally distributed through these hospitals to health centres and priority is naturally given to the hospitals before health centres are given their share of medical supplies or equipment. Similarly communities near health centres are more likely to have better services than those near community health services because health centres are responsible for rural community supplies and equipment. More research, close monitoring, and frequent audits are needed to improve the management and handling of medical supplies and services between different levels of the health delivery system in Chikwawa.

Finally this study is not without its limitations. Data was based on retrospective reporting by the most senior woman in each household. This may have created biases due to incomplete responses, and unrepresentative individual data. During the survey mothers were not given a precise definition of what constitutes an episode of diarrhoea. Therefore, questions relied on the mother’s perception of the disease other than clinical or actual definitions. This may have created variations among different households and communities because perception of an illness episode is not the same across different groups of people. To reduce the effect of these methodological limitations, questionnaires from each enumerator were carefully audited after each day’s survey and the data was screened to ensure consistency of approach to questioning and responses and to determine if the data conformed to expected patterns.

The survey required mothers to recall information of their previous behaviour in seeking treatment for a child or a family member. There was a risk that some diarrhoeal episodes would not be recalled due to long time when the illness occurred. However, since the aim of the study was essentially to understand factors that influence care seeking behaviour for diarrhoeal illness, this risk was overlooked on the basis that the information obtained would outweigh the discrepancies in forgotten diarrhoeal episodes. Moreover, recall bias is reported to be related to level of women’s education, with more educated women most likely to remember and distinguish most illnesses, therefore controlling for women’s education in the analysis may have captured a large part of the self-selective nature of reporting [27,28].

## 7. Conclusions

Despite limitations of the study, these results have an important role to play in developing health policy at regional and Government level and the promotion of health delivery in Chikwawa district. The results clearly show that significant number of families prescribe to the use of ORS or rush to the health facility for case management of diarrhoea. While this is the case, it is important to note that the administration of ORS has been shown to be associated with several problems in families due to problems in understanding written instructions and the administration of the correct concentration of ORS (i.e., *w*/*v*)—as recommended by WHO [29]. More research is needed in Chikwawa to assess handling and preparation of ORS and to observe behaviour when there is diarrhoeal illness at household level and within communities. With regard to visits to health facilities it is important to note that other studies have observed that those who visit health facilities sometimes do so (i) as a last resort after they have tried home treatment; (ii) after persistence in illness; or (iii) after illness is already at a serious stage [30]. This study has also documented people’s complaints about absence of health personnel and qualified staff at health facilities and lack or shortages of drugs. The study has further documented people’s complaints about transport and distance problems to health facilities. What is needed is a comprehensive concept addressing several dimensions, ranging from: (a) management and proper coordination of delivery of resources and services especially between different levels of the Chikwawa health delivery system; (b) availability of adequate healthcare workers at all levels of the Chikwawa health delivery system [31,32]; (c) affordability and accessibility of healthcare resources and services to all communities; (d) acceptability and quality of care; and (e) the intensification of health education messages on: (i) the use and management of ORS; (ii) what constitutes prompt treatment with regard to diarrhoeal illness; and (iii) the concept of diarrhoea illnesses. Furthermore, there is need for further research to: (i) examine the operations and performance of the different levels of healthcare delivery system with the aim of gaining a deeper understanding of the measures that can improve healthy delivery—not only in Chikwawa district, but the country as well; (ii) compare the cost of ORS and other fluids from the market or vendors against the cost of transport or transport problems to health facilities; (iii) examine the socio-economic factors that influence the decision making process of people who choose to provide or not to provide ORS and (iv) investigate factors that encourage or demoralise people to access health facilities for treatment of diarrhoea.

## Figures and Tables

**Table 1:** Summary measures for outcome variables on diarrhoeal care seeking behaviour.

Variable	Frequency	(%)
*Action normally taken when a household member has diarrhoea*	N = 1403	
Visits a health facility	940	67.0
Administers ORS obtained from health facility	220	15.7
Administers ORS bought from shop, market or vendors	151	10.8
Administers other fluids plus doing nothing	92	6.6
*Action that was taken when a child had last diarrhoea*	N = 1059	
Administered (ORS)	717	67.9
Administered other fluids	146	13.8
Visited health facility	136	12.9
None	57	5.4

Notice that any respondent could give more than one answer to these questions i.e., these were questions with multiple answers.

**Table 2:** Summary measures for predictor variables on diarrhoeal care seeking behaviour.

Variable	N = 1403		(%)	
***Categorical variables***				
*School*				
None	558		39.8	
Primary	757		54.0	
At least Secondary	88		6.3	
*Relative wealth*				
Low	512		36.5	
Medium	458		32.6	
High	433		30.9	
*Distance to nearest health facility*				
<1 km	491		35.0	
1 to 2 km	592		42.2	
>2 km	320		22.8	
HSA *				
Yes	959		68.4	
No	444		31.6	
VHC **				
Yes	944		67.3	
No	459		32.7	
*Problems to reach health facility*				
Long distance or transport costs	**1020**		**72.7**	
Too much work	91		6.5	
Other reasons	**115**		**8.3**	
Problems at health facility *				
Cost of medical services	174		12.4	
Long waiting time or health workers don’t show	**1019**		**72.6**	
No drugs	496		35.4	
Other	**105**		**7.5**	
*Time taken to treat a diarrhoea patient*				
Same day	1190		84.8	
One day	**126**		**9.0**	
Two or more day	87		6.2	
*Continuous variables*	*mean*	*median*	*minimum*	*maximum*
Maternal age ***	35.00	31.00	15	89
Household diarrhoea endemicity	0.486	0.200	0.00	5.000
Household size	5.59	5	1	13

* HSA—Health surveillance assistant, ** VHC—Village Health Committee, *** maternal age here refers to the age of the most senior woman in the household.

**Table 3:** Multilevel logistic regression models to identify determinants of household care-seeking behaviour when a family member has diarrhea.

Variables	Action after a Member of Household Has Diarrhoea Illness						Action after a Member of Household Has Diarrhoea Illness					
	Visit a Health Facility			Home Use of ORS from Hospital			Home Use of Bought ORS			Home Use of Other Fluids or Nothing		
	OR	(95% CI)	*p-*Value	OR	(95% CI)	*p-*Value	OR	(95% CI)	*p-*Value	OR	(95% CI)	*p-*Value
*School*												
None	(Reference group)											
Primary	0.698	(0.538, 0.914)	0.010 **	0.914	(0.644, 1.297)	0.603	1.822	(1.197, 2.801)	0.005 **	1.105	(0.684, 1.786)	0.674
Secondary	0.387	(0.230, 0.651)	0.000 **	1.649	(0.869, 3.127)	0.124	2.586	(1.234, 5.366)	0.012 **	1.682	(0.619, 4.572)	0.308
*Relative Wealth*												
Low	(Reference group)											
middle	0.905	(0.684, 1.209)	0.518	0.869	(0.600, 1.257)	0.452	2.034	(1.310, 3.158)	0.002 **	0.631	(0.368, 1.094)	0.100 *
high	0.932	(0.691, 1.259)	0.661	1.083	(0.748, 1.553)	0.686	1.492	(0.932, 2.411)	0.097 *	0.631	(0.368, 1.083)	0.098 *
*Any HSA?*												
Yes	(Reference group)											
No	1.083	(0.827, 1.419)	0.565	0.794	(0.554, 1.127)	0.202	1.162	(0.771, 1.733)	0.476	1.02	(0.613, 1.682)	0.947
*Any VHC?*												
Yes	(Reference group)						(Reference group)					
no	0.896	(0.670, 1.197)	0.452	1.01	(0.684, 1.477)	0.971	1.221	(0.794, 1.859)	0.362	0.951	(0.583, 1.568)	0.855
*Distance to nearest health facility*												
<1 km	(Reference group)											
1 km to 2 km	0.914	(0.533, 1.568)	0.74	0.65	(0.350, 1.197)	0.168	1.492	(0.794, 2.829)	0.212	1.84	(1.010, 3.320)	0.045 **
>2 km	1.062	(0.560, 1.994)	0.863	0.477	(0.225, 1.010)	0.053 *	1.568	(0.756, 3.254)	0.23	1.733	(0.878, 3.387)	0.114
*Maternal age*	0.99	(0.980, 1.000)	0.046 **	1.003	(0.990, 1.010)	0.617	1	(0.990, 1.010)	1	1.02	(1.000, 1.030)	0.061 *
*HH DRR*	0.565	(0.383, 0.844)	0.005 **	1.297	(1.067, 1.584)	0.009 **	0.97	(0.756, 1.259)	0.831	1.094	(0.810, 1.477)	0.574
*HH size*	0.896	(0.835, 0.970)	0.006 **	1.03	(0.951, 1.116)	0.435	1.02	(0.923, 1.116)	0.749	1.127	(1.010, 1.259)	0.035 **
	$\sigma_{u}^{2}$	(95% CI)	*p-*value	$\sigma_{u}^{2}$	(95% CI)	*p-*value	$\sigma_{u}^{2}$	(95% CI)	*p-*value	$\sigma_{u}^{2}$	(95% CI)	*p-*value
*CE (u*_0*j*_*)*	0.28	(0.025, 0.54)	0.031 **	0.38	(−0.01, 0.75)	0.053 *	1.31	(0.896, 1.915)	0.161	1.054	(0.896, 1.246)	0.528

CI—Credible interval; HH—Household; CE—Community effects; HH DRR—Household endemicity; HSA—Health surveillance assistant; VHC—Village health committee; OR—Odds ratio; * *p* ≤ 0.10; ** *p* ≤ 0.05.

**Table 4:** Multilevel logistic regression models to identify determinants of household care-seeking behaviour when a child had diarrhea.

Variables	Action after a Child Had Diarrhoea Illness						Action after a Child Had Diarrhoea Illness					
	Visit a Health Facility			Home Use of ORS			Home Use of Other Fluids			Traditional Methods or Nothing		
	OR	(95% CI)	*p-*Value	OR	(95% CI)	*p-*Value	OR	(95% CI)	*p-*Value	OR	(95% CI)	*p-*Value
*School*												
None	(Reference group)											
Primary	0.698	(0.454, 1.072)	0.100 *	1.234	(0.914, 1.665)	0.178	1.083	(0.719, 1.632)	0.708	0.741	(0.402, 1.363)	0.333
Secondary	0.56	(0.221, 1.419)	0.223	1.568	(0.835, 2.945)	0.162	1.221	(0.533, 2.829)	0.633	0.097	(0.007, 1.363)	0.084 *
*Relative Wealth*												
Low	(Reference group)											
middle	1.336	(0.835, 2.117)	0.23	0.869	(0.589, 1.139)	0.246	1.259	(0.819, 1.915)	0.302	0.779	(0.394, 1.537)	0.468
high	1.433	(0.878, 2.340)	0.148	0.951	(0.670, 1.363)	0.801	0.844	(0.522, 1.363)	0.496	0.844	(0.419, 1.682)	0.626
*Any HSA?*												
Yes	(Reference group)											
No	1.185	(0.771, 1.822)	0.432	0.861	(0.631, 1.173)	0.349	1.094	(0.719, 1.649)	0.685	1.02	(0.549, 1.896)	0.94
*Any VHC?*												
Yes	(Reference group)											
no	1.507	(0.951, 2.363)	0.081 *	0.705	(0.507, 0.970)	0.035 **	1.419	(0.932, 2.138)	0.104	0.878	(0.463, 1.682)	0.706
*Distance to nearest health facility*												
<1 km	(Reference group)											
1 km to 2 km	1.336	(0.650, 2.718)	0.431	0.741	(0.463, 1.197)	0.223	1.616	(0.933, 2.801)	0.086 *	0.577	(0.284, 1.173)	0.132
>2 km	2.054	(0.942, 4.482)	0.072 *	0.512	(0.304, 0.869)	0.014 **	2.117	(1.150, 3.857)	0.015 **	0.491	(0.210, 1.162)	0.108
*Maternal age*	0.98	(0.970, 1.000)	0.018 **	0.99	(0.980, 1.000)	0.11	1.02	(1.000, 1.030)	0.015 **	1.02	(1.000, 1.041)	0.046 **
*HH DRR*	1.185	(0.923, 1.522)	0.188	0.99	(0.819, 1.197)	0.927	0.869	(0.664, 1.139)	0.324	0.869	(0.565, 1.323)	0.513
*HH size*	0.98	(0.878, 1.094)	0.689	1.072	(0.990, 1.150)	0.081 *	0.97	(0.878, 1.094)	0.689	0.896	(0.771, 1.051)	0.178
	$\sigma_{u}^{2}$	(95% CI)	*p-*value	$\sigma_{u}^{2}$	(95% CI)	*p-*value	$\sigma_{u}^{2}$	(95% CI)	*p-*value	$\sigma_{u}^{2}$	(95% CI)	*p-*value
*CE (u*_0*j*_*)*	0.35	(−0.11, 0.82)	0.131	0.14	(−0.07, 0.35)	0.205	0.09	(−0.14, 0.32)	0.456	0.11	(−0.20, 0.41)	0.498

CI—Credible interval; HH—Household; CE—Community effects; HH DRR—Household endemicity; HSA—Health surveillance assistant; VHC—Village health committee; OR—Odds ratio; * *p* ≤ 0.10; ** *p* ≤ 0.05.

## References

[1] Institute for Health Metrics and Evaluation GBD Publications 2016. http://www.healthdata.org/gbd/publications.

[2] National Statistical Office (NSO), and ORC Macro (2005). Malawi Demographic and Health Survey 2004.

[3] World Health Organization (WHO) (2006). Country Health System Fact Sheet 2006, Malawi.

[4] United Nations Millennium Development Goals. http://www.un.org/millenniumgoals/.

[5] Department of Planning, Ministry of Health (2004). The Joint Programme of Work for Sector Wide Approach.

[6] Simwaka B.N., Bello G., Banda H., Chimzizi R., Squire B.S.B., Theobald S.J. (2007). The Malawi National Tuberculosis Programme: An equity analysis. Int. J. Equity Health.

[7] D’Souza R.M. (2003). Role of health seeking behaviour in child mortality in the slums of Karachi, Pakistan. J. Biosoc. Sci..

[8] Ellisa A.A., Winch P., Daou Z., Gilroy K.E., Swedberg E. (2006). Home management of childhood diarrhoea in southern Mali—Implications for the introduction of zinc treatment. Soc. Sci. Med..

[9] Masangwi S.J., Morse T.D., Ferguson N., Zawdie G., Grimason A.M. (2008). A preliminary analysis of the Scotland-Chikwawa Health Initiative Project on morbidity. Mag. Int. Fed. Environ. Health.

[10] Muirhead R.W., Davies-Colley R.J., Donnison A.M., Nagels J.W. (2004). Faecal bacterial yields in artificial flood events: Quantifying in-stream stores. Water Res..

[11] Kandala N.B., Magadi M.A., Madise N.J. (2006). An investigation of district spatial variations of childhood diarrhoea and fever morbidity in Malawi. Soc. Sci. Med..

[12] National Statistical Office (NSO), UNICEF (2008). Malawi Multiple Indicator Cluster Survey 2006.

[13] Gwatkin D.R., Rustein S., Johnson K., Pande R.P., Wagstaff A. (2000). Socio-Economic Differences in Health, Nutrition, and Population in Bangladesh. http://www1.worldbank.org/prem/poverty/health/data/bangladesh/bangladesh.pdf.

[14] Leyland A.H., Goldstein H. (2001). Multilevel Modelling of Health Statistics.

[15] Fielding A., Yang M., Goldstein H. (2003). Multilevel ordinal models for examination grades. Stat. Model..

[16] Rasbash J., Steele F., Browne W., Prosser B. (2004). A User's Guide to MLwiN.

[17] Goldstein H. (2003). Multilevel Statistical Models.

[18] Browne W.J. (2003). MCMC Estimation in MLwiN.

[19] Masangwi S.J., Morse T.D., Ferguson N., Zawdie G., Grimason A.M., Namangale J.J. (2009). Behavioural and environmental determinants of childhood diarrhoea in Chikwawa, Malawi. Desalination.

[20] Manda S.O.M. (1999). Birth intervals, breastfeeding, and determinants of childhood mortality in Malawi. Soc. Sci. Med..

[21] Pongou R., Ezzati M., Salomon J.A. (2006). Household and community socioeconomic and environmental determinants of child nutritional status in Cameroon. BMC Public Health.

[22] Osumanu I.K. (2007). Household environmental and behavioural determinants of childhood diarrhoea morbidity in the Tamale Metropolotan Area (TMA), Ghana. Dan. J. Geogr..

[23] Rufener S., Mäusezah D., Mosler H., Weingartner R. (2010). Quality of drinking-water at source and point-of-consumption—Drinking cup as a high potential recontamination risk: A field study in Bolivia. J. Health Popul. Nutr..

[24] Blum L.S., Oria P.A., Olson C.K., Breiman R.F., Ram P.K. (2011). Examining the use of oral rehydration salts and other oral rehydration therapy for childhood diarrhea in Kenya. Am. J. Trop. Med. Hyg..

[25] Government of Malawi (2002). National Malaria Control Programme.

[26] Zere E., Moeti M., Kirigia J., Mwase T., Kataika E. (2007). Equity in health and healthcare in Malawi: Analysis of trends. BMC Public Health.

[27] Filmer D. (2005). Fever and its treatments among the more poor and less poor in sub-Saharan Africa. Health Policy Plan..

[28] Kazembe L.N., Muula A.S., Appleton C.C., Simoonga C. (2009). Joint spatial modelling of common morbidities of childhood fever and diarrhoea in Malawi. Health Place.

[29] Forsberg B.C. (2007). Diarrhoeal Diseases in Low- and Middle-Income Countries: Trends, Management and Control. Ph.D. Thesis.

[30] Nyamongo I.K. (1990). An Introduction to the Medical History of Ethiopia.

[31] Masangwi S.J., Grimason A.M., Morse T.D., Ferguson N.S., Kazembe L.N. (2012). Community knowledge variation, bed-net coverage, the role of a district healthcare system, and their implications for malaria control in southern Malawi. S. Afr. J. Epidemiol. Infect..

[32] Hockin A., EWB Water and Sanitation Team The Role of Health Surveillance Assistants in Community-Led Total Sanitation in Malawi. http://www.communityledtotalsanitation.org/sites/communityledtotalsanitation.org/files/Role_of_HSAs.pdf.

